# Complete chloroplast genome of *Chloranthus henryi* (chloranthaceae)

**DOI:** 10.1080/23802359.2019.1637793

**Published:** 2019-09-12

**Authors:** XueDie Liu, XingYu Liao, ZhongJian Liu, SiRen Lan

**Affiliations:** a Key Laboratory of National Forestry and Grassland Administration for Orchid Conservation and Utilization, College of Forestry, Fujian Agriculture and Forestry University, Fuzhou, Fujian, China;; b Fujian Colleges and Universities Engineering Research Institute of Conservation and Utilization of Natural Bioresources, College of Forestry, Fujian Agriculture and Forestry University, Fuzhou, Fujian, China

**Keywords:** *Chloranthus henryi*, plastid genome, phylogeny, Chloranthaceae

## Abstract

The complete chloroplast genome of *Chloranthus henryi*, an important traditional Chinese herbal medicine, was sequenced and characterized in this study. The genome size is 157,990 bp in length with 37.3% GC content. Two inverted repeats of 26,151 bp are separated by a large single-copy region (87,148 bp), and a small single-copy region (18,569 bp). A total of 131 genes were identified, including 86 protein-coding genes, 37 tRNA genes and 8 rRNA genes. Eight plastome accessions from Chloranthales, Austrobaileyales, Nymphaeales, and Amborellales were selected to assess the phylogenetic placement of genus.

*Chloranthus* is a small and well-defined genus of the Chloranthaceae (Kong et al. 2002). Diagnostic characteristics of this genus include a suffrutescent or herbaceous habit, terminal or axillary inflorescence, small bisexual and perianthless flowers (Kong et al. 2002). It consisted of 15 species with relatively primitive morphological characters placed in a basal angiosperm family, Chloranthaceae (Chloranthales). Many species in the genus were used as important medicinal plants in China and other countries (Zhang et al. 2015). Therefore, we assembled and annotated the complete chloroplast genome of *Chloranthus henryi*. It will provide potential genetic resources for elucidating possible evolutionary relationships between *C. henryi* and other basal angiosperms (Doyle and Endress 2015).

The plant sample was collected from Wuyishan National Nature Reserve (117°40′36.96″E, 27°45′22.09″ N), Fujian Province of China, and voucher specimen deposited at Herbarium of College of Forestry, Fujian Agriculture and Forestry University (specimen code FAFU08019). We assembled the complete cp genome of *C. henryi* and total genomic DNA extracted from fresh leaves of *C. henryi*, with 350 bp randomly interrupted by the Covaris ultrasonic breaker (Covaris, East Sussex, UK) for library construction. The constructed library was sequenced PE150 by Illumina Hiseq Xten platform (Illumina, San Diego, CA), approximately 2GB data was generated. Illumina data was filtered by script in the cluster (default parameter: -L 5, -p 0.5, -N 0.1). Plastid genome was assembled by GetOrganelle pipe-line (https://github.com/Kinggerm/GetOrganelle), and plastid-like reads were obtained, and the reads were viewed and edited by Bandage (Wick et al. 2015). The sequence of *C. henryi* assembly was performed with GENEIOUS R11.15 (Biomatters Ltd., Auckland, New Zealand) (Kearse et al., 2012). The assembled genome was annotated using DOGMA (Wyman et al. 2004) coupled with manual corrections for start and stop codons. The annotation result was drawn with the online tool OGDRAW (http://ogdraw.mpimp-golm.mpg.de/) (Lohse et al. 2013).

The complete plastid genome sequence of *C. henryi* (GenBank accession MK922064) was 157,990 bp in length, with a large single-copy (LSC) region of 87,148 bp, a small single-copy (SSC) region of 18,569 bp, and a pair of inverted repeats (IR) regions of 26,151 bp. A total of 131 gene species were annotated, including 86 protein-coding (PCG), 37 transfer RNA (tRNA), and 8 ribosomal RNA (rRNA) gene species. The complete genome GC content was 37.30%. In order to reveal the phylogenetic position of *C. henryi*, a phylogenetic analysis was performed based on 9 complete cp genomes of living mesangiosperms (*Chloranthus spicatus*, *Chloranthus japonicas*, *Sarcandra glabra*, *Nymphaea colorata*, *Brasenia schreberi*, *Kadsura longipedunculata*, *Schisandra chinensis*) and one taxon (*Amborella trichopoda*) as outgroups, they were all downloaded from NCBI GenBank. The sequences were aligned by MAFFT v7.388 (Katoh and Standley 2013), and phylogenetic tree constructed by RAxML (Stamatakis et al. 2014) (Figure 1). The relationship between *Chloranthus* and *Sarcandra* was strongly supported (bootstrap = 100%). The maximum-likelihood phylogenetic analysis shows that *C. henryi* was closely related to *C. spicatus*.

**Figure 1.**
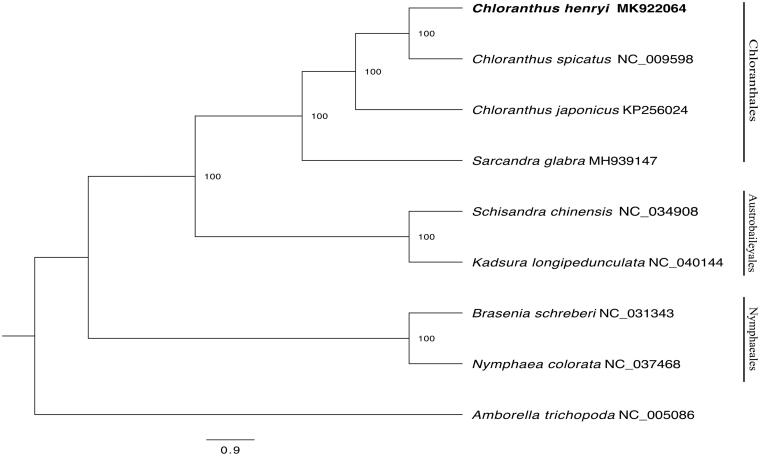
RAxML phylogeny of *C. henryi* based on 9 complete cp genomes (Bootstrap values are shown above each node, and a “_” indicates 100% statistical support value).
